# Supplementary material for: Exploring DNA methylation profiles in the pathogenesis of human osteoporosis via whole-genome bisulfite sequencing
Source: PLoS One. 2026 Jul 16;21(7):e0341108. doi: 10.1371/journal.pone.0341108 (PMC13374884; doi:10.1371/journal.pone.0341108)
Supplement: S2 Table — (DOCX) [file pone.0341108.s002.docx]

**Supplementary Table S6. Sensitivity analysis: methylation changes of six candidate genes after adjusting for sequencing depth.**

| Gene | No. of CpGs in region | Mean log₂FC (adjusted) | Min raw P value | Median adjusted P value | Direction |
| --- | --- | --- | --- | --- | --- |
| MSX1 | 17 | 1.128 | 0.00053 | 0.343 | Hypermethylated |
| HOXD4 | 65 | 0.568 | 0.0132 | 0.612 | Hypermethylated |
| AXIN2 | 5 | 0.857 | 0.00180 | 0.396 | Hypermethylated |
| WNT5A | 197 | 0.600 | 0.00050 | 0.537 | Hypermethylated |
| TGFB1 | 5 | -1.061 | 0.00071 | 0.332 | Hypomethylated |
| STAT3 | 6 | -0.693 | 0.00695 | 0.405 | Hypomethylated |

**Legend:** Linear model (limma) adjusted for sequencing depth. Mean log₂FC is the average log₂ fold change (osteoporosis vs. control) across CpGs within each original DMR. None of the median adjusted P values were < 0.05. Direction indicates hypermethylation (positive) or hypomethylation (negative) in osteoporosis patients.
