# Supplementary material for: Exploring DNA methylation profiles in the pathogenesis of human osteoporosis via whole-genome bisulfite sequencing
Source: PLoS One. 2026 Jul 16;21(7):e0341108. doi: 10.1371/journal.pone.0341108 (PMC13374884; doi:10.1371/journal.pone.0341108)
Supplement: S3 Table — (DOCX) [file pone.0341108.s003.docx]

**Table S3. Genomic annotation of differentially methylated regions for the six prioritized genes**

| Gene | Chromosome | DMR start (hg19) | DMR end (hg19) | Length (bp) | Annotation (relative to gene) | CpG island context | Overlap with regulatory elements (ENCODE) |
| --- | --- | --- | --- | --- | --- | --- | --- |
| MSX1 | chr4 | 4865917 | 4866048 | 132 | TTS (transcription termination site) | CpG island (Dist.to.CGI = 0)¹ | Not annotated (hg19) |
| HOXD4 | chr2 | 177016376 | 177016548 | 173 | exon 1 of 2 (promoter proximal) | CpG island (overlap) | Not annotated (hg19) |
| AXIN2 | chr17 | 63554635 | 63554707 | 73 | exon 2 of 2 | CpG island (Dist.to.CGI = 0)¹ | Not annotated (hg19) |
| WNT5A | chr3 | 55518324 | 55518397 | 74 | intron 2 of 5 | CpG island shore (dist = –384 bp) | Not annotated (hg19) |
| TGFB1 | chr19 | 41832484 | 41832566 | 83 | intron 2 of 3 | CpG island (Dist.to.CGI = 0)¹ | Not annotated (hg19) |
| STAT3 | chr17 | 40489722 | 40489800 | 79 | intron 6 of 22 | Open sea (dist = –24774 bp) | Not annotated (hg19) |

¹ Dist.to.CGI from the DMR analysis output. For MSX1, AXIN2 and TGFB1, the reported distance to the nearest CpG island is 0, but manual inspection of genomic coordinates (e.g., for MSX1: DMR 4,865,917–4,866,048 vs. nearest CGI 4,866,438–4,866,813) indicates that the DMR does not physically overlap the CGI; the software assignment may reflect the presence of an unannotated CGI or a threshold effect. Therefore, these DMRs are annotated conservatively as “CpG island (dist=0)” based on the computational output.
