# Supplementary figures and images for: Exploring DNA methylation profiles in the pathogenesis of human osteoporosis via whole-genome bisulfite sequencing
Source: PLoS One. 2026 Jul 16;21(7):e0341108. doi: 10.1371/journal.pone.0341108 (PMC13374884; doi:10.1371/journal.pone.0341108)

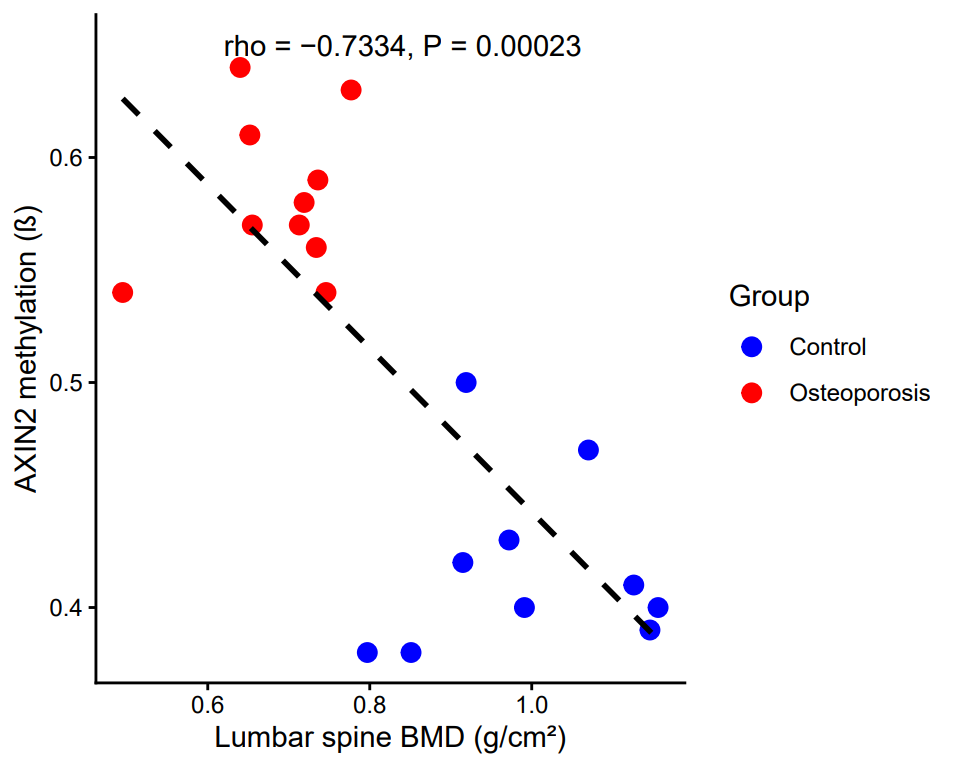

Supplement: S1 Fig — (TIFF) [file pone.0341108.s004.tiff]

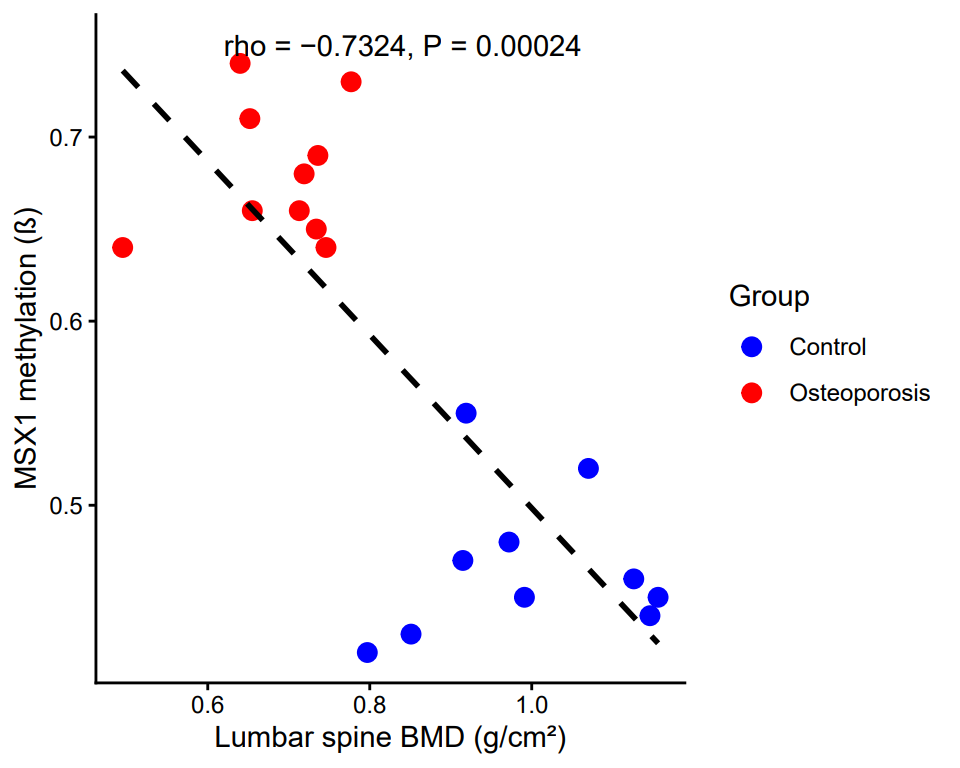

Supplement: S2 Fig — (TIFF) [file pone.0341108.s005.tiff]

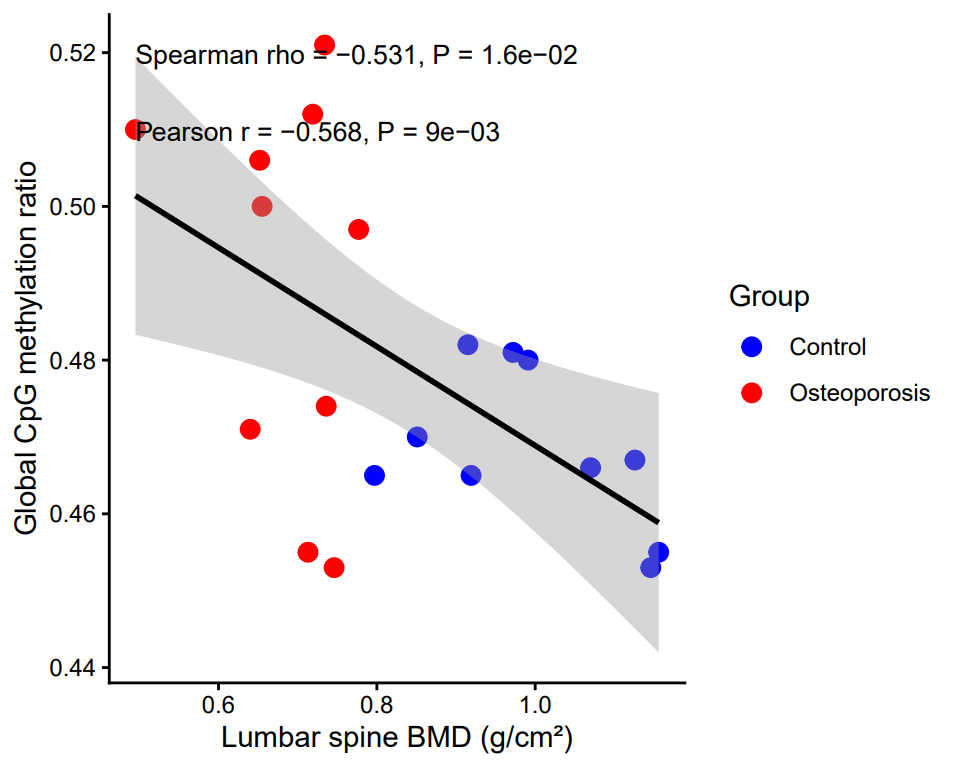

Supplement: S3 Fig — (Spearman rho = −0.531, P = 0.0159; Pearson rho = −0.567, P = 0.0090). Each dot represents one participant. (TIFF) [file pone.0341108.s006.tiff]

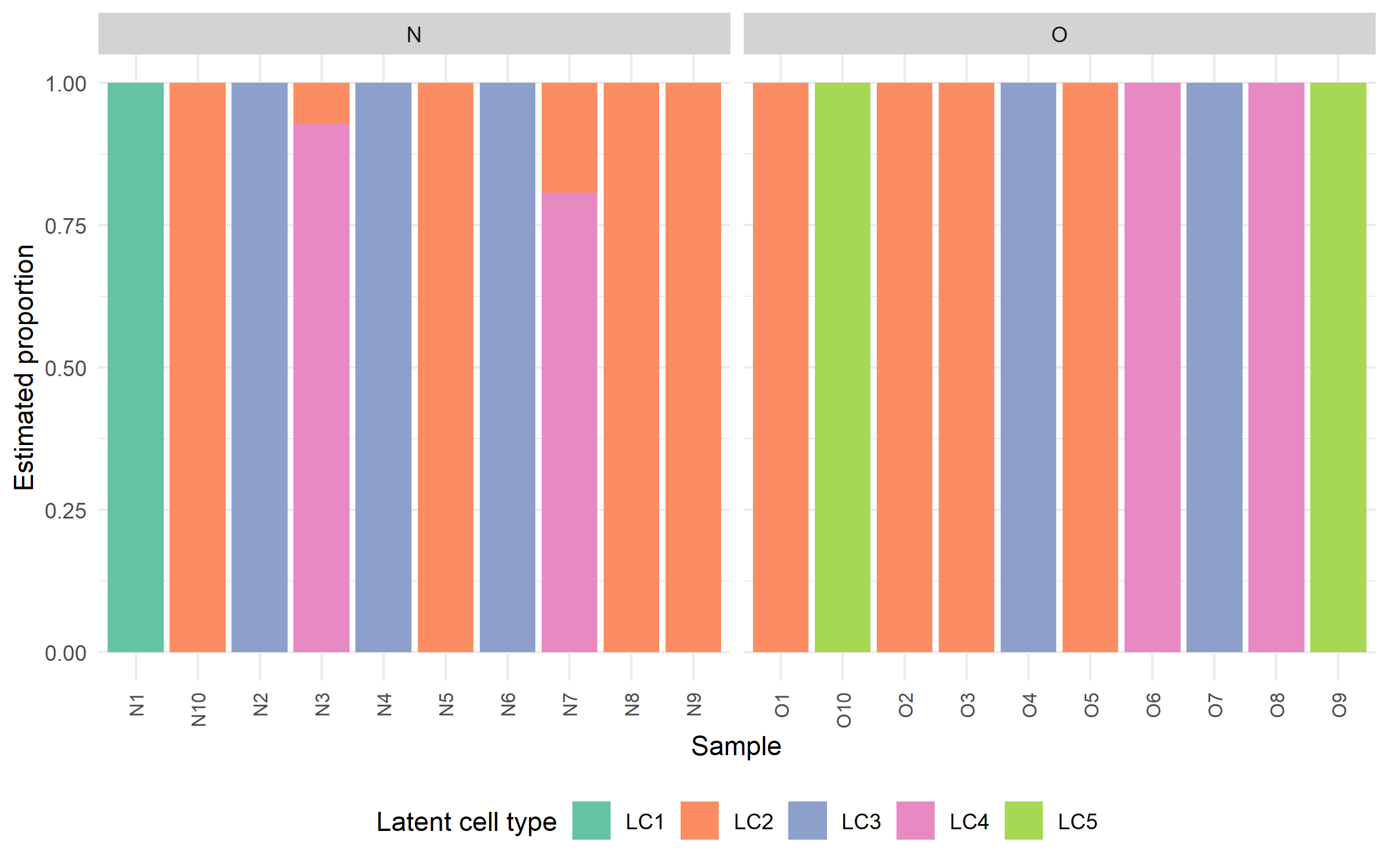

Supplement: S4 Fig — Most samples show a dominant component (>0.99), indicating that the algorithm did not infer continuous mixtures. (TIFF) [file pone.0341108.s007.tiff]
